# First approach to pod dehiscence in faba bean: genetic and histological analyses

**DOI:** 10.1038/s41598-020-74750-1

**Published:** 2020-10-19

**Authors:** David Aguilar-Benitez, Inés Casimiro-Soriguer, Ana M. Torres

**Affiliations:** IFAPA Center Alameda del Obispo, Apdo 3092, 14080 Córdoba, Spain

**Keywords:** Agricultural genetics, Plant breeding, Plant genetics

## Abstract

Pod dehiscence causes important yield losses in cultivated crops and therefore has been a key trait strongly selected against in crop domestication. In spite of the growing knowledge on the genetic basis of dehiscence in different crops, no information is available so far for faba bean. Here we conduct the first comprehensive study for faba bean pod dehiscence by combining, linkage mapping, comparative genomics, QTL analysis and histological examination of mature pods. Mapping of dehiscence-related genes revealed conservation of syntenic blocks among different legumes. Three QTLs were identified in faba bean chromosomes II, IV and VI, although none of them was stable across years. Histological analysis supports the convergent phenotypic evolution previously reported in cereals and related legume species but revealed a more complex pattern in faba bean. Contrary to common bean and soybean, the faba bean dehiscence zone appears to show functional equivalence to that described in crucifers. The lignified wall fiber layer, which is absent in the paucijuga primitive line Vf27, or less lignified and vacuolated in other dehiscent lines, appears to act as the major force triggering pod dehiscence in this species. While our findings, provide new insight into the mechanisms underlying faba bean dehiscence, full understanding of the molecular bases will require further studies combining precise phenotyping with genomic analysis.

## Introduction

Faba bean (*Vicia faba* L.) is a world-wide cultivated grain legume, known for its high protein content and yield potential. Its ability to grow under a wide range of climates and soil types has made faba bean one of the preferred crops for agricultural production^1^, being the second most yielding feed grain legume after soybean (*Glycine max* L.)^2^. Besides diseases and pests, pod dehiscence is one of the major constraints on faba bean production in dry areas^3^. In some legumes such as common vetch (*Vicia sativa* L.) cultivars, dehiscence rate of mature pods can reach 40 to 60%^4, 5^. In soybean, Bhor^6^ reported 50 to 100% yield losses in susceptible cultivars under severe dry climate conditions. In faba bean, yield losses at harvest can be substantial as well, and according to GRDC^7^, pod dehiscence can reach 30% maturity.

Pod dehiscence refers to the shattering of the pod shell, which enables the successful shattering of seeds^5^. A crucial strategy for seed dispersal in wild species ranks among the main sources of yield loss in cultivated crops and likely was one of the first traits selected against during crop domestication. Valve aperture during dehiscence is mediated by environmental factors and physical forces in the tissue, which in turn are determined by the properties of the wall cells around the valve suture. Relative humidity and variations in temperature trigger hygroscopic tensions in the pod walls, which are mediated by the different mechanical properties of lignified and non-lignified tissues and by changes in turgor associated to fruit maturation^5, 8^.

Even after genetic selection against dehiscence, some degree of dehiscence remains in cultivated crops. Since the dominance of the trait difficult its efficient introgression into cultivated lines, dehiscence is still a key target trait in plant breeding programs. Genetic analysis of pod dehiscence has been conducted in different crop legumes, such as soybean, common bean (*Phaseolus vulgaris* L.), cowpea (*Vigna unguiculata* L.), pea (*Pisum sativum* L.), common vetch, lentil (*Lens culinaris* Medikus), azuki bean (*Vigna angularis* L.) and lupin (*Lupinus angustifolius* L.)^9^ but not faba bean. In these species, dehiscence is controlled by one or two dominant genes or by quantitative trait loci (QTL). A syntenic region, controlling the trait was identified in pea and lentil^10, 11^, suggesting that orthologous genes may be modified during the domestication of these two crops.

Extensive results on the genetic control of dehiscence have been reported in cereals and in the Brassicaceae model species *Arabidopsis thaliana*. In cereals seed dehiscence is controlled by genes encoding transcription factors, indicating that differences in seed dispersal are associated with changes in transcriptional regulation^12^. In *Arabidopsis*, forward genetic approaches identified several transcription factors as well^13^, although it is unclear whether they are conserved in legumes^9^.

**Figure 1:**
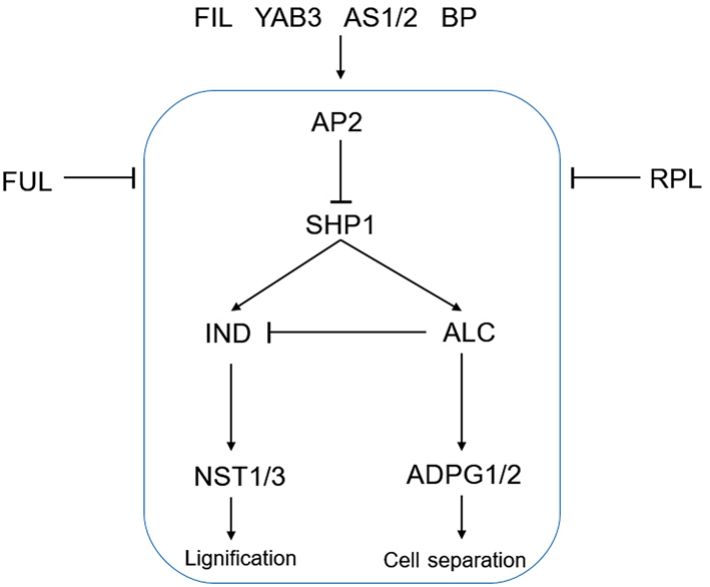
Schematic diagram of the genetic pathway in the dehiscence zone (DZ). Modified from Ballester and Ferrándiz 2017.

The crucifer family produces siliques, a pod structure consisting of two fused valves (carpels), connected by a distinct edge or replum. In contrast, the legume pod has a single folded seed-bearing carpel lacking a replum. Another important structure delimiting the region between the valves and the replum is the dehiscence zone (DZ), which should be lignified in order to allow the separation of the valve from the replum. Differences in the DZ lead either to indehiscence or dehiscence of a plant. Ballester and Ferrándiz^14^ updated the genetic network directing morphogenesis of the DZ in *Arabidopsis* and reported the transcription factors and enzymatic functions with major roles in DZ formation. Thus, *NAC SECONDARY WALL THICKENING PROMOTING FACTOR 1/3* (*NST1/3*) promotes lignification while *ARABIDOPSIS DEHISCENCE ZONE POLYGALACTURONASE 1* (*ADPG1/2*) allows cell separation (Fig. 1). *ADPG1/2* is controlled by the action of *ALCATRAZ* (*ALC*), a negative regulator of the *INDEHISCENT* (*IND*) gene, which in turn promotes the expression of *NST1/3*. Furthermore, both *ALC* and *IND* expression is both regulated by *SHATTERPROOF 1* (*SHP1*). The *shp* and *ind* mutants lack lignification and are fully indehiscent, thus corroborating the importance of DZ lignification in pod dehiscence^15–17^.

Upstream of these genes, *APETALA2* (*AP2*), represses the expression of *SHP1*. Adjacent to the DZ tissues, two important genes downregulate the expression of all dehiscence pathway-related genes: *FRUITFULL* (*FUL*) in the valve and *REPLUMLESS* (*RPL*) in the replum^17, 18^. Other transcription factors regulating the expression of valves, DZ and replum are *FILAMENTOUS FLOWER* (*FIL*), *YABBY3* (*YAB3*), *ASYMMETRIC LEAVES1/2* (*AS1/2*) or *BREVIPEDICELLUS* (*BP*) (Fig. 1).

Compared with model species, knowledge on the molecular control of dehiscence in legumes is limited. Despite the lack of a replum, the DZ is very similar showing a lignified separation layer along the pod between the two valves^19, 20^. This indicates that valve aperture is triggered by the same physical mechanism, although the lack of the replum tissue points towards a different anatomical basis.

In legumes, the molecular basis of indehiscence has been investigated in detail in soybean^21, 22^. A major QTL, PDH1 (QTL for Pod Dehiscence 1), encoding a dirigent-like protein involved in lignin biosynthesis, was reported as candidate for dehiscence regulation. The indehiscent genotype, *pdh1*, carries a premature stop codon and lacks lignin depositions along the valve. *PDH1* was shown to promote pod dehiscence by increasing the twisting force in the pod wall, the driving force of pod dehiscence^21^. The transcription factor *WRKY12* was also described to promote lignin biosynthesis and cell wall deposition in sorghum, *Medicago truncatula* and *Arabidopsis*^23, 24^*.*

Besides lignin deposition, cellulose and hemicellulose content in cowpea was related to the ability to resist pod opening^25^. In common bean, high dehiscence levels were correlated with high carbon and lignin contents of the pod valves^26^, while a transcriptomic study in *V. sativa* revealed candidate genes involved in the biosynthesis of different cell wall components^27^. Recently, a candidate gene approach in *Cicer arietinum,* using a RIL population derived from an interspecific cross, identified a major QTL (*PDH1*) regulating pod dehiscence in combination with the regulatory genes *FUL*, *ALC* and *AP2* previously described in *Arabidopsis*^28^. Pod morphology (e.g. pod length) also affects the degree of dehiscence. For example, short pods in *Brassica* and *Sinapis* provide an indehiscent phenotype by reducing physical stress on the dehiscence zone^29, 30^.

Here we conducted a comprehensive investigation of pod dehiscence in faba bean by combining linkage mapping of candidate genes, QTL analysis and histological analysis of pod valves. A set of orthologs of dehiscence related genes described in *A. thaliana*, *M. truncatula*, *P. sativum* and *C. arietinum* were tested for polymorphism and further mapped in a segregating population. The final objectives were to: (1) saturate the faba bean map with candidate genes controlling dehiscence, (2) compare the locations of the faba bean QTLs with those reported in related legumes species and (3) perform a comparative histological analysis in pods from contrasting lines. Our results add to the understanding of the genetic basis of dehiscence and will help to identify candidate genes responsible for indehiscence in this agronomically important crop.

## Results

### Phenotypic evaluation of dehiscence

**Figure 2:**
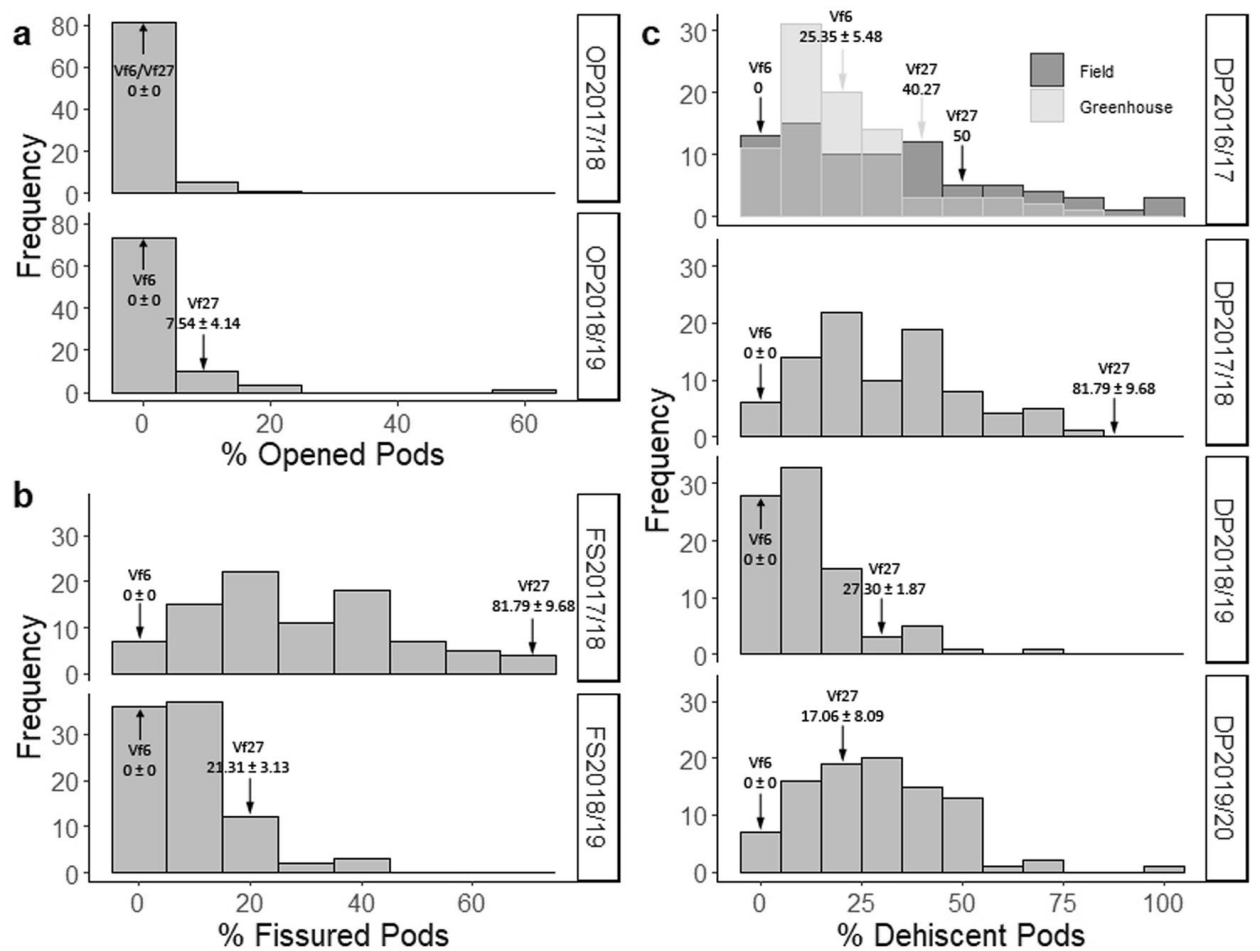
Frequency distribution of dehiscence traits recorded in the Vf6 x Vf27 RIL population. (**a**) Opened Pods (OP) in 2017/18 and 2018/19; (**b**) Fissured Pods (FS) in 2017/18 and 2018/19; (**c**) Dehiscent Pods (DP) in 2016/17, 2017/18, 2018/19 and 2019/20. The phenotypic values (mean ± SE) of the parental lines are indicated by arrows.

Phenotypic trait analysis showed a continuous distribution of dehiscence (Fig. 2), suggesting that the character is controlled by multiple genes in this population. The frequency distribution of all variables did not fit the normal distribution (*P* < 0.05). In the four years, a positive skewness was observed (Fig. 2), indicating a transgressive segregation towards lower pod dehiscence values contributed by the female line Vf6.

Table 1 shows the mean phenotypic values and some basic descriptive statistics for the dehiscence traits recorded: opened pods (OP), fissured pods (FS) and dehiscent pods (DP). Parental lines showed significant differences (*P* < 0.05) for FS in 2017/18 and 2018/19, and DP in 2017/18 and 2018/19. Except in OP2017/18, line Vf27 exhibited higher phenotypic values than Vf6 for all the dehiscence traits. Evaluation in greenhouse (DPG) in 2016/17 showed no statistical differences between the parental lines. DP and FS showed a wider range of variation than OP. The range of OP in the different seasons varied from 0 to > 65% (Table 1).

**Table 1:** Phenotypic values of completely opened pods (OP), fissured pods (FS) and dehiscent pods in greenhouse (DPG) or field (DPF) for the parental lines and the RIL population Vf6 x Vf27 in 2016/17, 2017/18, 2018/19 and 2019/20.

Trait	Vf6	Vf27	Range (Min–Max)	Mean (± SE)
OP2017/18	0 ± 0	0 ± 0	0–16.67	0.86 ± 0.32
OP2018/19	0 ± 0	7.54 ± 4.14	0–65.15	2.99 ± 0.86
FS2017/18*	0 ± 0	81.79 ± 9.68	0–74.07	29.96 ± 1.99
FS2018/19*	0 ± 0	21.31 ± 3.13	0–39.86	9.23 ± 0.97
DPG2016/17	25.35 ± 5.48	40.27	0–82.60	20.63 ± 1.90
DPF2016/17	0	50	0–100	32.80 ± 2.92
DP2017/18*	0 ± 0	81.79 ± 9.68	0–81.61	31.25 ± 2.03
DP2018/19*	0 ± 0	27.30 ± 1.87	0–67.38	12.66 ± 1.37
DP2019/20	0 ± 0	17.06 ± 8.09	0–100	29.42 ± 1.83

Significant differences between parental lines (*P* ≤ 0.05) are indicated by asterisks.

Correlations among traits were evaluated across the four years at *P* < 0.05 and *P* < 0.01. As summarized in Table 2, the highest correlations were found between DP and FS in 2017/18 (0.98) and 2018/19 (0.88), followed by DP and OP in 2018/19 (0.58). Except for DP2019/20, DP values in the greenhouse showed moderate but significant correlations ranging from 0.25 to 0.37 with the rest of the DP field ratings. There were also significant correlations between 0.21 and 0.31 among the DP field values except between DP2018/19 with DPF2016/17 and DP2017/18. No correlation was detected among the OP field values in 2017/18 and the DP data collected in the other three years, except for DPG2016/17. A strong attack of aphids during the flowering and podding period in 2018/19 and the high incidence of broomrape (*Orobanche crenata*) in 2017/18 might be the main reasons for the lack of correlation between the traits recorded (Table 2).

**Table 2:** Correlations between the dehiscence traits scored.

	OP2017/18	OP2018/19	FS2017/18	FS2018/19	DPG2016/17	DPF2016/17	DP2017/18	DP2018/19	DP2019/20
OP2017/18	1.00								
OP2018/19	0.14	1.00							
FS2017/18	-0.01	0.12	1.00						
FS2018/19	– 0.15	0.23*	0.21	1.00					
DPG2016/17	0.30**	0.35**	0.19	0.21	1.00				
DPF2016/17	– 0.07	0.08	0.31**	0.16	0.37**	1.00			
DP2017/18	0.10	0.13	0.98**	0.19	0.25*	0.31**	1.00		
DP2018/19	– 0.05	0.58**	0.17	0.88**	0.26*	0.11	0.17	1.00	
DP2019/20	-0.19	0.11	0.22*	0.17	0.07	0.31**	0.21*	0.22*	1.00

Significant correlations are indicated in bold. Asterisks: *P ≤ 0.05; **P ≤ 0.01.

ANOVA results for the genotype x environment (G x E) analyses of each evaluated trait are shown in Supplementary Table 1. For all traits, significant differences were obtained between Genotype (RILs) and Environment (years of evaluation). For OP and DP evaluations, significant differences were also obtained for the G x E interaction, suggesting that pod opening differed for a certain genotype across the years.

### Dehiscence candidate genes and marker development

Fifty one dehiscence-related candidate genes (Supplementary Table 2) were selected on the basis of current knowledge of their roles in pod dehiscence control from different plant species, including *Arabidopsis thaliana* (27 genes), *V. sativa* (22), *G. max* (1) and *Solanum lycopersicum* (1). Gene sequences were subjected to BLAST searches to identify orthologs in other sequenced legume species closely related to faba bean (*C. arietinum, M. truncatula* and *P. sativum*) and positive blast matches for all of them were found (Supplementary Table 2).

Based on these sequences, primers were designed and used for amplification with DNA from *V. faba*. We obtained amplification products for 26 *A. thaliana* and 16 V*. sativa* gene markers. The gene marker from *S. lycopersicum* (Vf_TAGL1) also produced an amplification product, whereas attempts to amplify the Vf_PDH1 marker from *G. max* were unsuccessful. Primer sequences, size of the amplified DNA fragments and annealing temperatures for each candidate gene are shown in Supplementary Table 3.

The sequences of the amplified DNA fragments were aligned to identify possible Single Nucleotide Polymorphisms (SNPs) between the parental lines. A total of 38 amplified sequences (23 candidates from *A. thaliana*, 14 from *V. sativa* and one from *S. lycopersicum*) contained SNPs in restriction enzyme recognition sites and were thus transformed to Cleavage Amplified Polymorphisms (CAPs). Ten of the detected SNPs did not contain cleavage sites for available restriction enzymes and therefore internal primers were designed for these sequences. Where the internal primers failed, SNPs were genotyped using the MassArray iPLEX (Sequenom) platform (https://www.cegen.org). Only one of the amplified genes (Vf_TAGL1) could not be genotyped in the population.

Finally, 44 out of 163 genes genotyped using the KASPar technique, displayed polymorphisms between the parental lines and were included in the genetic map.

### Genetic map and synteny analysis

**Figure 3:**
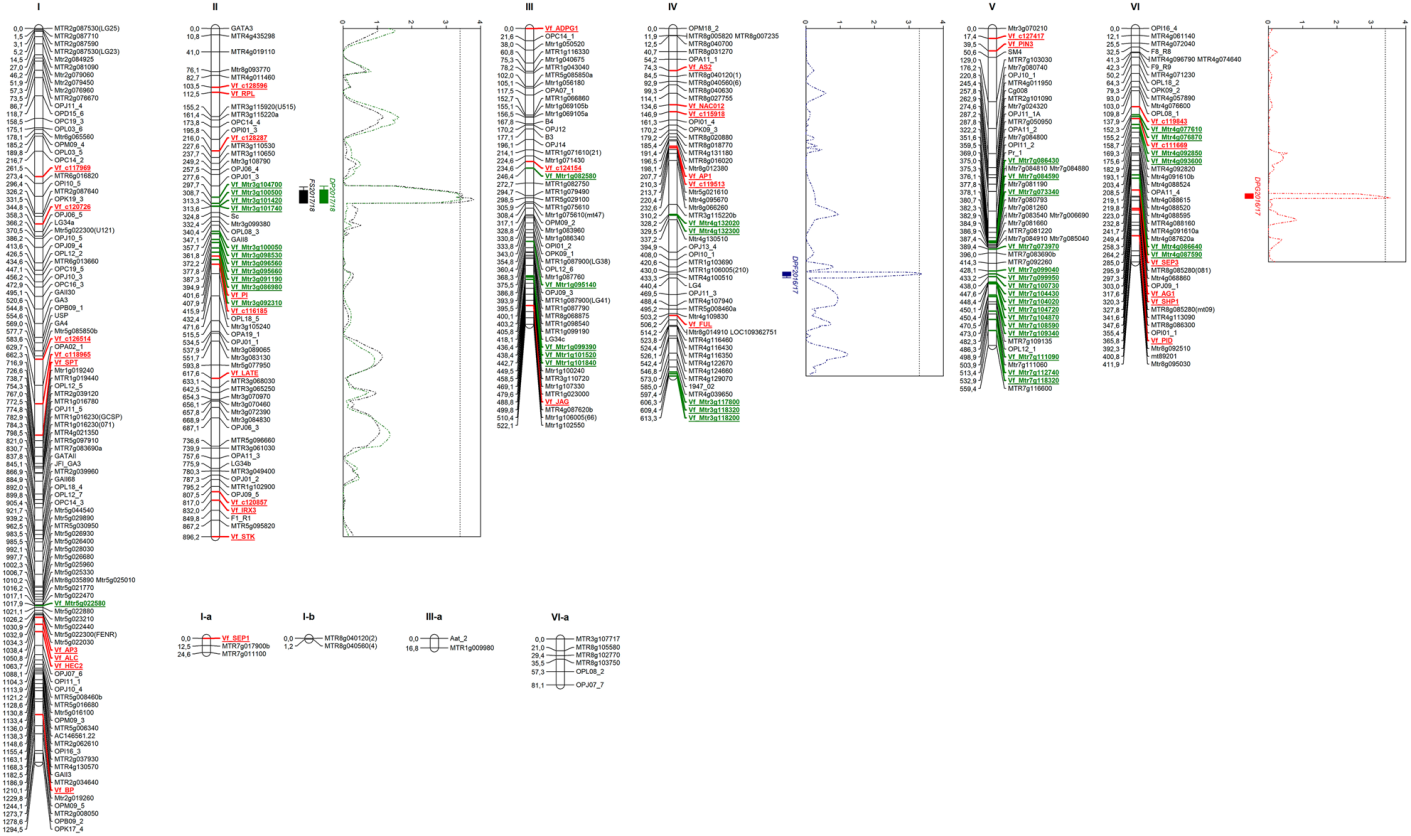
Linkage map and QTLs for dehiscence traits detected in the Vf6 x Vf27 RIL population. QTL locations are represented with bars (2-LOD interval) and boxes (1-LOD interval). Candidate gene markers are in red. Molecular markers used for map saturation by the Kompetitive Allele Specific PCR (KASP) assay are in green. FS, Fissured pods; DPG, Dehiscent pods in the greenhouse; DPF; Dehiscent pods in the field.

To build a more saturated map in the RIL population, segregation data from 44 KASPar markers and 37 dehiscence-related markers were combined with a previous data set^31^. Four of the later markers deviated significantly (*P* < 0.05) from the expected Mendelian inheritance ratio of 1:1 (Table 3). Vf_AG1, Vf_SEP3 and Vf_SHP1 were skewed towards the Vf27 allele whereas Vf_c128596 was in favour of the Vf6 allele. The linkage map consists of six main linkage groups and 5 fragments, and spans nearly 4.421,1 cM (Fig. 3). The existence of common markers with previous consensus maps^32, 33^ allowed to assign all the LGs to the corresponding faba bean chromosomes (chrs.). All candidate-gene markers except Vf_YAB3 were included in the new genetic map. The number of candidate-gene markers in each LG ranged from one in LGI to nine in LGII, and the distorted markers Vf_AG1, Vf_SEP3 and Vf_SHP1 mapped together in the distal part of chr. VI.

**Table 3:**
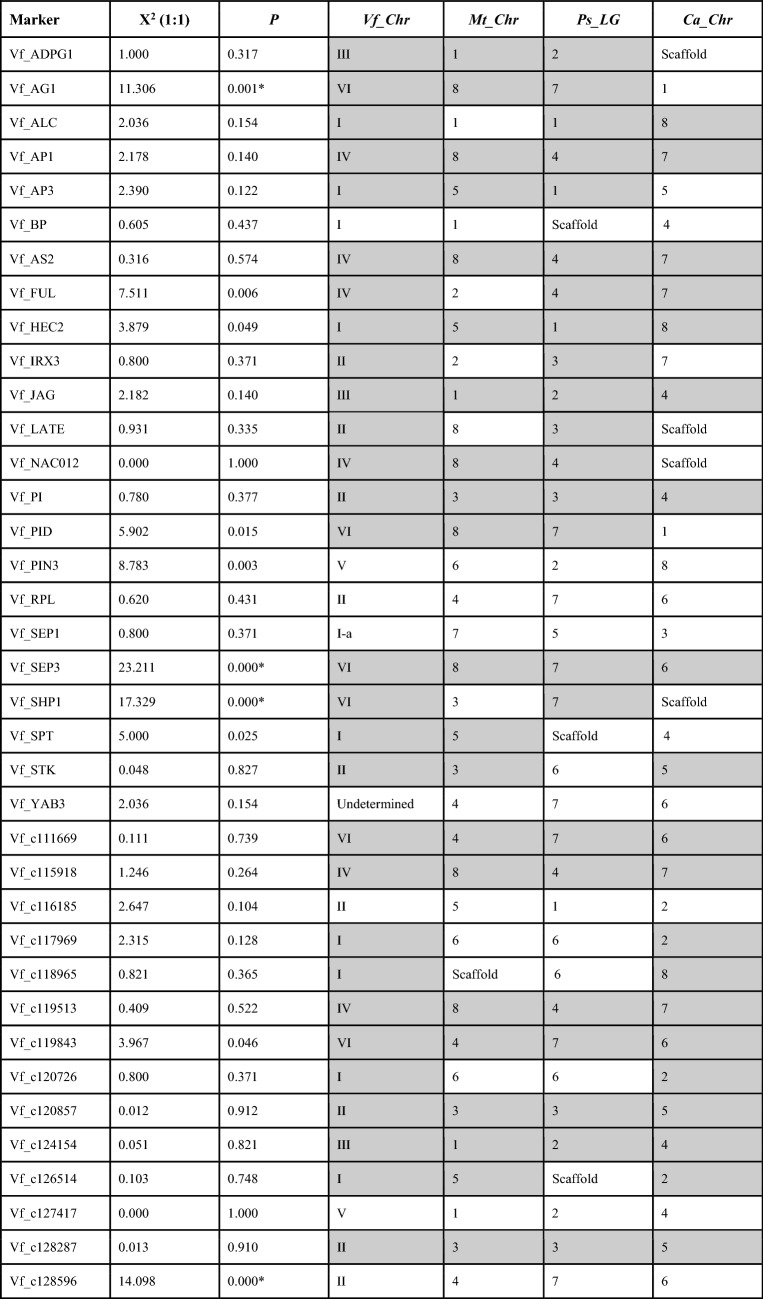
Genetic segregation, chi-square test and location of the dehiscence related gene markers in faba bean (Vf) and related legume species: Mt, *Medicago truncatula*; Ps, *Pisum sativum*; Ca, *Cicer arietinum*.

Grey cells indicate syntenic chromosomes (Chr) and linkage groups (LG). Absence of fit to the expected 1:1 segregation is indicated by an asterisk.

In order to determine if these genes maintain their relative positions in the genomes of different legumes, we determined synteny between *V. faba* and the related species *M. truncatula, P. sativum* and *C. arietinum* (Table 3). The level of synteny was high (78%), with 29 out of 37 gene markers showing conservation in at least one other species. Twenty of markers were syntenic with *C. arietinum*, 21 with *M. truncatula* and 23 with *P. sativum*. Finally, thirteen markers (Vf_AP1, Vf_AS2, Vf_HEC2, Vf_JAG, Vf_PI, Vf_SEP3, Vf_c111669, Vf_c115918, Vf_c119513, Vf_c119843, Vf_c120857, Vf_c124154 and Vf_c128287), exhibited conserved synteny across all four species, preserving the co-localization in the homologous chromosomes.

### QTL analysis

Three significant QTL regions (LOD threshold > 3.3) in chromosomes II, IV and VI were related to dehiscence resistance (Table 4, Fig. 3). Except for QTL DPG2016/17, the additive effects in most of the associated markers were negative, indicating that the resistance enhancing alleles originated from the dehiscence resistant parent Vf6. The QTL in chr. II (FS2017/18) close to marker Vf_Mtr3g104700 explained 20.7% of the phenotypic variation, whereas the QTL of DP2017/18 accounted for 18.5% of the phenotypic variation in the same region.

**Table 4:** Trait name, peak position (cM), chromosome (Chr), flanking markers, LOD scores, additive effects and phenotypic variation explained (R^2^) by the QTLs for dehiscence resistance detected in the Vf6 x Vf27 RIL population.

Trait	Peak	Chr	Flanking markers	LOD	Additive effects	R^2^
FS2017/18	301.705	II	Vf_Mtr3g104700	3.8	− 8.51046	20.7
DP2017/18	300.705	II	Vf_Mtr3g104700	3.48	− 8.20145	18.5
DPF2016/17	432.986	IV	Mtr4g100510	3.38	− 10.5056	15.2
DPG2016/17	290.958	VI	Vf_SEP3/Mtr8g085280(81)	3.51	8.26058	18.6

Another QTL for resistance to dehiscence (DPF2016/17) was detected in chr. IV, explaining 15.2% of the variation. The DPF2016/17 peak position corresponds to the marker Mtr4g100510. Finally, QTL DPG2016/17 was detected in chr. VI, flanked by the candidate gene for dehiscence SEP3, and for Mtr8g085280(81), explaining 18.6% of the phenotypic variation. The 2019/20 evaluation, although showing a moderate correlation with the dehiscence traits scored in the previous seasons, did not reveal any significant QTL region.

A BLASTp search was performed using *Arabidopsis* as a model, to identify the gene orthologs flanking each QTL and the results are shown in Supplementary Table 4.

### Histological characterization

**Figure 4:**
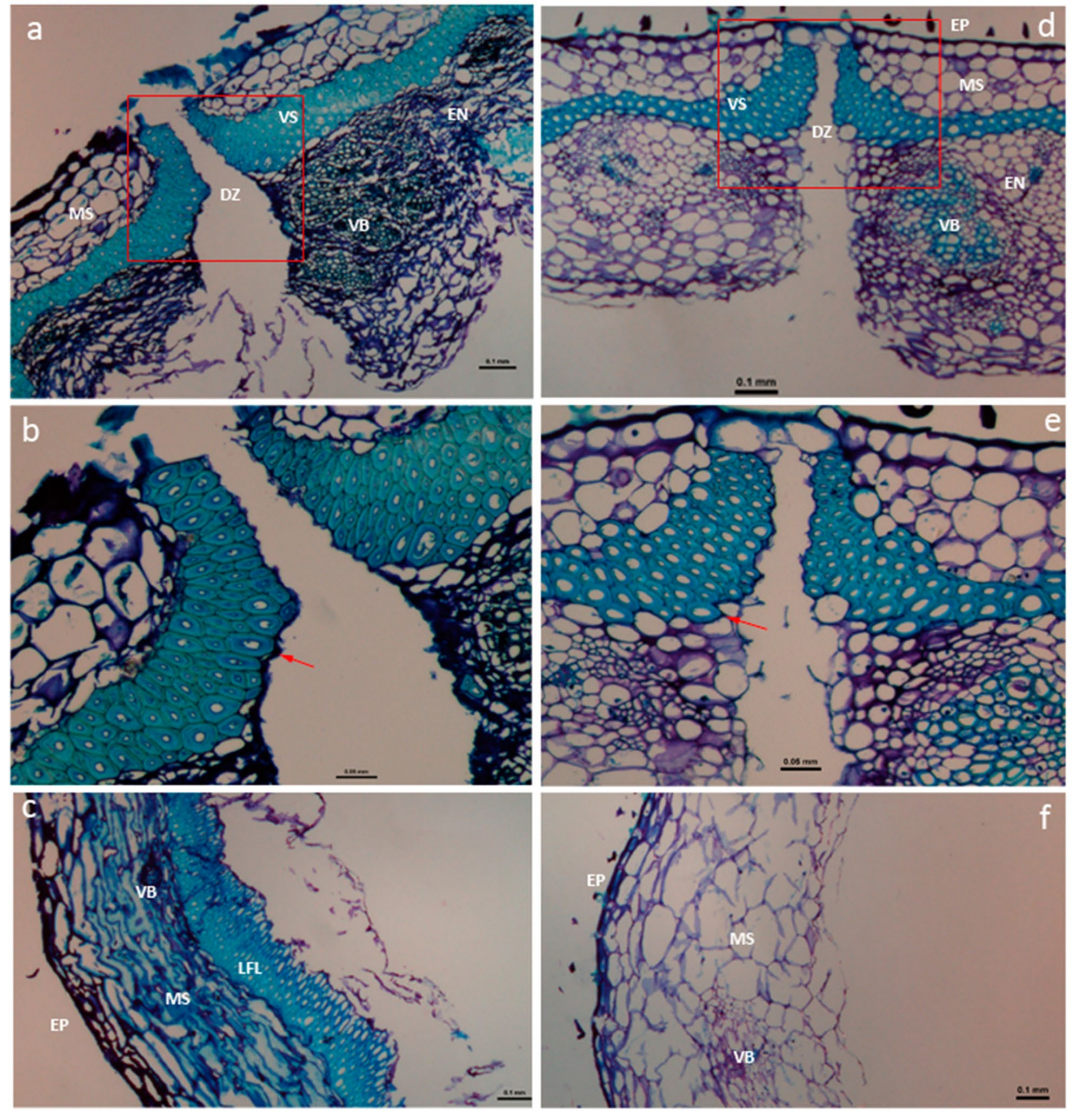
Histological study of pods in the parental faba bean lines VF6 (left) and Vf27 (right). (**a**) Cross-sections of the distal part ventral suture in Vf6 and Vf27 (**d**). Detail of the dehiscence zone in Vf6 (**b**) and Vf27 (**e**); red arrows indicate differences in the degree of lignification of the VS cell wall. Transverse section of the lateral side of the pod in Vf6 (**c**) and Vf27 (f). Bars, 0.1 mm (**a**,**c**,**d**,**f**); 0.05 mm (**b**, **e**). DZ, dehiscence zone; EP, epidermis; MS, mesocarp; VS, ventral sheath; VB, vascular bundle; EN, endocarp; LFL, lignified fiber layer.

Samples obtained for histological analysis consisted of proximal and distal transverse cuts corresponding to the ventral and dorsal sutures of mature pods. The micrographs of the transverse sections revealed that the faba bean pod wall (pericarp) follows the common arrangement and structure described in legume crops (Fig. 4). The exocarp consist of a single-layered epidermis (EP) while the mesocarp (MS) is arranged in several layers of parenchyma. The endocarp (EN) is composed of a sclerenchyma which heavily thickened by lignin in dorsal and ventral sutures (or sheath), with two lateral branches extending into the seed coats and a vascular bundle (VB) containing the xylem and phloem tissues. Both exocarp and mesocarp are rich in pectins, as indicated by metachromatic staining with toluidine blue (Fig. 4). The DZ tissue between valves almost disappeared after the inclusion in paraffin, probably due to the maturity of the samples.

Histological staining revealed clear differences between parental lines, regarding both the area and the perimeter of the respective lignified cell layer (Table 5) and the proportion of lignin deposition within cells (Table 6). In all samples, the indehicent parent Vf6 showed a higher sheath lignified area (stained in light blue) than the dehiscent line Vf27 (Fig. 4, Table 5). In the proximal dorsal zone, this measurement was performed in total stained tissue since there was no clear distinction between left and right valves. In this case, the central zone in Vf6 also contained more cells than in Vf27 (Supplementary Fig. 1 ).

**Table 5:** Area and perimeter of the sheath lignified layers in the parental lines, measured at the distal and proximal sides of the ventral and dorsal pod sutures.

		Right		Left	
		Area (mm^2^)	Perimeter (mm)	Area (mm^2^)	Perimeter (mm^2^)
Dista zone / ventral suture	Vf6	0.101	2.412	0.124	2.604
	Vf27	0.058	1.847	0.053	2.002
Distal zone / dorsal suture	Vf6	0.060	1.510	0.069	1.635
	Vf27	0.016	0.883	0.017	0.827
Proximal zone / ventral suture	Vf6	0.091	2.779	0.086	2.254
	Vf27	0.048	1.751	0.051	1.91
Proximal zone / dorsal suture*	Vf6	0.117	3.609		
	Vf27	0.037	2.041		

Lignified cells arranged as a single layer without left–right distinction are indicated by an asterisk.

**Table 6:** Percentage of lignin cell occupation in the faba bean parental lines Vf6 and Vf27, measured in different pod zones.

	Distal ventral	Distal dorsal	Proximal ventral	Proximal dorsal
Vf6	**91.88 ± 0.008**	**87.74 ± 0.010**	71.92 ± 0.024	**76.87 ± 0.020**
Vf27	**79.13 ± 0.014**	**68.24 ± 0.014**	73.39 ± 0.012	**69.44 ± 0.010**

Significant differences according to ANOVA are indicated in bold (*P* < 0.01).

Cell walls in the sheath of the pods were heavily thickened (Fig. 4). Nevertheless, the lumen of the cells appeared to be almost occluded in the indehiscent Vf6 line as compared of the dehiscent pods in the Vf27 parent (Fig. 4b,e). Except for the proximal ventral zone, which is less relevant for dehiscence, the percentage of lignin deposition inside the cells was higher in Vf6 than in Vf27 (*P* < 0.01). Vf6 showed higher lignin deposition in the distal zone than in the proximal zones, both at ventral and dorsal sutures, whereas in the dehiscent line Vf27 lignin deposition was similar between the two zones (Table 6).

Interestingly, the two parental lines exhibited a marked difference in the geometrical arrangement and histological characteristics of the lateral cell wall (Fig. 4c,f). The three pericarp layers EP, MS and EN with a continuous ring of lignified cells, were clearly distinguishable in the dehiscent resistant Vf6 line, while in Vf27 only the EP could be distinguished and no lignin deposition was found in any of the other layers.

## Discussion

Pod dehiscence causes important agronomic losses and regarded as a key trait in crop domestication^22, 34^. In legumes such as chickpea, soybean or pea, clear differences in dehiscence between wild (dehiscent) and cultivated (indehiscent) accessions were reported^22, 35, 36^, showing the results of many years of domestication. In faba bean, however, the wild progenitor is unknown, making it difficult the comparison between a wild dehiscent genotype and modern varieties.

Here we conducted the first comprehensive study of pod dehiscence in faba bean by combining comparative genomics, linkage mapping of candidates genes, QTL analysis and histological examination of pod valves from a segregating population. To this aim, we used a RIL population derived from the contrasting lines Vf6 (indehiscent) and Vf27 (dehiscent), the latter being a primitive paucijuga form found from Afghanistan to India, whose phenotype is similar to the hypothetical wild progenitor^37^.

Pod dehiscence in faba bean was characterized in four consecutive years under field and greenhouse conditions, based on previous data obtained from model plants and related legume species. Phenotypic evaluation of dehiscence showed a gradient similar to that described in *P. vulgaris*^26^, ranging from pods completely indehiscent and pods with valves separated to some degree, to fine fissured valves and completely open valves (twisted and non-twisted). Three QTLs were identified although none of them was significant across all seasons, as would be expected for a trait which is strongly influenced by environmental conditions such as humidity, temperature, duration of pod drying and biotic stresses^38, 39^.

The lack of significance for some QTLs in 2018/19 can be further attributed to a severe aphid attack leading to complete exploitation of the hosts plant. As a result, the pods failed to develop correctly and the phenotypic evaluation was somewhat distorted. Similarly, the QTL results obtained in 2017/18 could be explained by a severe broomrape (*Orobanche crenata*) attack. This parasitic plant is widespread in Mediterranean areas and west Asia, lacks chlorophyll and depends on the host plant for nutrition. The broomrape attack in 2017/18 affected plant and pod development in the critical phase of biomass accumulation, leading to abortions and a marked decrease in pod number. Thus, biotic stresses influencing the genetic effects could, together with the environmental conditions, represent the main reasons for the observed lack of strong correlation between dehiscence traits and growing seasons, corroborated by the G x E significance observed in some of the traits evaluated.

To identify candidate genes co-localizing with QTLs for faba bean dehiscence resistance, we selected a set of candidate marker genes involved in pod dehiscence in *Arabidopsis* and other relevant crop species, which were found to be conserved between mono and dicotyledonous plants^38, 40^. As a consequence, these shared domestication-related loci have become common targets for genetic studies and breeding programs. Here we surveyed 51 faba bean orthologs of dehiscence-related genes from *A. thaliana, V. sativa, G. max* and *S. lycopersicum*, in order to unveil the presence of homologous pathways and loci controlling dehiscence. BLAST searches and PCR analysis with designed primers identified 43 orthologs, which retained enough sequence similarity to be amplified in faba bean. Thirty eight of these contained SNPs and 37 were finally included in the new genetic map. The chromosomal positions of these markers among legume species was highly conserved, thus corroborating that collinearity allows to predict gene position across members of the same family.

QTL analysis revealed three significant QTLs for dehiscence, which were located in chromosomes II, IV and VI. The higher R^2^-value (20.7%) was obtained in the 2017/18 evaluation, when the dehiscence was evaluated as fissured pods. None of the major QTLs detected was stable across the environments, probably due to the biotic stresses mentioned above. This observation is supported by the lack of correlation between the respective dehiscence traits FS, OP and DP. Additional evaluations allowing more precise phenotyping in a controlled environment will be required to identify and validate QTLs consistent across different years.

Except for QTL DPG2016/17, which is flanked by Vf_SEP3 in chr. VI, none of the other QTLs showed a close relation with the dehiscence-related candidate gene markers. However, some of them exhibited synteny with previous QTLs detected in closely related species. For example, QTL DP2017/18 in chr. II, was syntenic with QTLs reported in *L. culinaris*^41^, *P. sativum*^10, 11^ and *M. truncatula*^36^. Similarly, QTLs in chr. IV and VI (DPF2016/17 and DPG2016/17) might correspond to syntenic blocks reported in *L. culinaris*^41^, *P. sativum*^11^, *C. arietinum*^28^ and *G. max*^21, 22, 42^ where other dehiscence related genes or QTLs were detected. As reported by Cannon^43^, most of the genes in papilionoid legume species are likely to be located in syntenic regions with respect to any other given papilionoid species. The results obtained here support these findings by confirming the conservation of large-scale synteny blocks for dehiscence regulation in orthologous chromosomal regions.

Important progress has been made in the characterization of the genetic basis of dehiscence in a number of model species and crops such as wheat, rice, soybean, beans, lentil, pea among others (reviewed by^5, 14, 26, 34, 38^). Collectively, these studies suggest that pathways and loci controlling pod dehiscence are broadly conserved across taxa, pointing towards homologous mechanisms underlying dehiscence. However, while a convergent phenotypic evolution was proposed across cereals, which might also have occurred at orthologous loci in closely related legume species, such evidence has not been obtained so far across more distant legume crops^34^. Comparative mapping between *P. vulgaris* and *G. max* suggested that the convergent evolution of the indehiscent phenotypes derived from mutations at different genes involved in pod cell wall biosynthesis and lignin deposition^44^, which suggested a more complex pattern in the genetic control of this trait in legumes.

In this study, a number of orthologs dehiscence-related genes from different species were genotyped and mapped to assess their role in faba bean indehiscence. Among these, only *SEP3*, encoding a MADS-box transcription factor, was found to flank a significant QTL for dehiscence resistance in 2016/17 (DP2016/17). MADS-box genes are known as key regulators of virtually every aspect of plant reproductive development and have served as important targets for selection during crop domestication^45^. *SEP3* was described as part of the ABCDE complex of floral organ determination^46–49^. In *Arabidopsis*, the C + E complex specifies carpels, being the class C gene *AGAMOUS* (*AG*)^50^ and the class E *SEPALLATA1/2/3/4* (*SEP1/2/3/4*)^51^. SEP3 interacts with genes regulated by auxin, gibberellic acid and brassinosteroids, including *PID* and *PIN*^46^. Immink^47^ described the interaction network of *SEP3*, showing the relation of this gene with the dehiscence pathway in *A. thaliana*^14^.

A SEPALLATA MADS-box protein from tomato was described as an activator of the abscission zone in the flower peduncle^52^. This protein forms a complex with JOINTLESS and MACROCALYX and activates other genes to transform meristem cells in a few layers of small cells (abscission zone). Auxin flow is also an important requisite for the formation of the dehiscence zone in *A. thaliana*^14^. Interestingly, our results revealed that QTL DPG2016/17 was also flanked by Mtr8g085280, an ortholog of the *Arabidopsis NO VEIN* gene that regulates *PIN* expression and auxin polarity^53^ thus suggesting that this process might also be involved in faba bean dehiscence resistance.

A recent transcriptomic analysis of dehiscence in *V*. *sativa* identified several genes related to cell wall modifications^27^, whose orthologs were analyzed in our study. However, none of these showed a significant correlation with dehiscence in *V. faba*. Moreover, some of the soybean and *Arabidopsis* pod dehiscence genes assayed here, such as *SHAT1-5*, *pdh1*, *SHP1*, *SHP2*, *IND,* and *ALC*, were not found to be differentially expressed in *V. sativa.* A similar lack of relation of *IND*, *SHP1* and *PDH1* with dehiscence, as observed in our study, was previously reported in a cross between yardlong bean and wild cowpea^25^.

Dehiscence in faba bean could be related with the expression level of certain genes. For example, fusion of the strong 35S promoter to the MADS-box gene *FUL* in *A. thaliana* resulted in overexpression of dehiscence-related genes *ALC*, *IND* and *STK*^54^. Similarity, overexpression of another MADS-box gene *AGL1* caused abnormal flowers and short, yellowish-green and early dehiscent pods, indicating a possible relationship between pod length and dehiscence^49^. Interestingly, the faba bean parental line Vf27 has short dehiscent pods, although we found no correlation between pod length and dehiscence traits (data not shown). Previous QTL analysis in our group^31, 32^ detected QTLs for pod length in chromosomes I and V, but none of them colocalized with the QTLs for dehiscence found in this work. This lack of coincidence suggest that pod length and dehiscence may not be related in faba bean.

Histological characterization of the faba bean pod sutures in mature pods revealed anatomical structures analogous to those described in other legumes such as soybean, common bean, pea or common vetch^22, 26, 30, 36, 55, 56^. Pod dehiscence arises from fissures that initiate in the ventral sutures, as recently observed in chickpea by Aguilar-Benitez^28^. Our micrographs from the pod opening sutures, show the progression of the valve separation and the collapse of cells in the DZ. Clear differences in the lignification pattern were detected between contrasted faba bean lines. Thus, although the cell walls in the sheath were heavily thickened in both parental lines, the cell lumen appeared to be almost occluded in the indehiscent Vf6 compared to the primitive dehiscent line Vf27. A major lignification in the DZ was reported to confer dehiscence resistance to mature pods in soybean^22^, *Brassica napus*^54^ and *P. vulgaris*^26, 55, 56^*.* Moreover, observation of the anatomical structure of pod ventral sutures in common vetch (*V. sativa*) revealed that all dehiscent vetches have abscission layers in the DZ that are absent in the indehiscent plants^57^. However, this finding, could not be corroborated in our faba bean sections, due to the advanced stage of maturation of the pod samples used in the study.

Clear-cut differences were observed between the two faba bean lines concerning the degree of lignification in the sclerenchyma inner cells of the pod walls. A continuous ring of cells with high lignification was specifically detected in the indehiscent line Vf6, while in Vf27 this lignified wall fiber layer (LFL) was absent. This observation is similar to that described in *Brassica* species, where indehiscent species show a more lignified endocarp than dehiscent ones^54^. By contrast, our findings are opposed to those reported from histological studies in common bean and soybean, where pod valves of the wild dehiscent lines showed a strong lignified wall fiber layer contrasting with a complete absence of lignin deposition in the indehiscent lines^21, 26, 34, 55, 56, 58^.

Because not all anatomical or histological differences between the primitive dehiscent line Vf27 and Vf6 are necessarily correlated with dehiscent traits, we analyzed the new faba bean dehiscent lines, 335, 756, 1068. Histological staining revealed a ventral sheath pattern similar to Vf27 with lower deposition of lignin compared to Vf6. However, a lignified wall fiber layer (LFL), which is absent in Vf27, was detected in these three dehiscent lines (Supplementary Fig. 2 ), although it was thinner and had an empty lumen. These results suggest the development of an intermediate, but correlated cell pattern from the primitive dehiscent phenotype Vf27 to the new dehiscent lines tested.

Although the tissue sections at this stage were difficult to obtain due to the fragility of the pod valves, we clearly observed an increased mechanical resistance of Vf6 compared with Vf27. Therefore we propose that the dehiscence zone of faba bean is functionally equivalent to that described in crucifers, although the underlying molecular mechanisms may differ. At the late stage of maturity, once lignification is completed and the valve attachment becomes weakened, the increased size of the vacuolated cells in the lateral walls of the dehiscent lines promote dehiscence. Loss of turgor due to desiccation of these vacuolated cells, coupled with reduced resistance to deformation caused by a lower level of lignin deposition could be the major force triggering pod dehiscence in the dehiscent faba bean lines.

In other plant species, pod dehiscence has also been linked to differences in turgor of the inner sclerenchymatic cell layer, as a result of dehydration^59–61^. Murgia proposed that lignification of the internal valve layer (LFL) contributes to dehiscence in soybean^26^. By contrast, our results in faba bean indicate that the absence of LFL in line Vf27 and the lower level of lignification in other dehiscent lines could reduce the stability of the valve, triggering cell detachment and separation of the valves after desiccation.

## Material and methods

### Plant material

A recombinant inbred line (RIL) population was used to determine the genomic regions associated with dehiscence. The population consists on 124 F8-F9 inbred lines obtained from the cross between the parental lines Vf6, an indehiscent equina type; and Vf27, a pod dehiscent, small seeded type (paucijuga), supposedly close to the putative wild faba bean progenitor^37, 62^. This population was previously used by our group to identify and validate QTLs controlling flowering and yield related traits^31^. For the histological studies three additional dehiscent lines (335, 756 and 1068) from the IFAPA faba bean core collection were included in the analysis.

### Phenotypic evaluation

The field evaluation was performed at Córdoba (Spain) along four agronomics seasons (2016/17, 2017/18, 2018/19 and 2019/2020). Meteorological data recorded for each agrononomic season are summarized in Supplementary Table 5. The RIL population was sown in November using a complete randomized design, with three to five plants per genotype and two replications. Only in 2016/17 pods from each line were evaluated twice in both, controlled and field condition. Mature pods were harvested and dried for 10 days at 20–40% relative humidity and 25 °C^10^ and then led to the extreme drying in the greenhouse. For the field scoring, one stem per plant was covered in the field with an isolation holey bag until the pods were completely dry. In the remaining seasons, plants were evaluated for dehiscence in the field when pods were dry.

Four pod categories were established: (1) indehiscent, (2) “fissured,” referring to pods with a fine open line between their valves but yet closed, (3) dehiscent with non-twisting valves, and (4) dehiscent with twisting valves. Because of the presence of intermediate cases in the same plant, in seasons 2017/18, 2018/19 and 2019/20, pod dehiscence was evaluated using three phenotypic traits: (1) percentage of opened pods, OP = (opened pods / total number of pods) × 100, (2) percentage of fissured pods, FP = (fissured pods / the total number of pods closed and/or fissured) × 100, excluding open pods, in an attempt to evaluate the trait independently and (3) percentage of dehiscent pods, DP = (opened and fissured pods / total number of pods) × 100. In 2016/17 and 2019/20, dehiscence was only evaluated as the percentage of dehiscent pods, with no distinction between completely open and fissured pods. Mean values for each parental line and RIL were calculated for each trait and replicate. Normality tests were performed to evaluate whether the data were normally distributed. ANOVA tests for Genotype x Environment analyses were performed. All statistical data analyses were carried out using the R software version 3.6.1^63^.

### Dehiscence candidate genes and primer design

A candidate gene strategy^64^ was applied for map enrichment and identification of functional candidates co-localizing with QTLs for dehiscence. Fifty one dehiscence-related genes were selected from different species (Supplementary Table 2): *Arabidopsis thaliana*^14, 38, 49, 65–67^*, G. max*^21^, *S. licopersicum*^68^ and *V. sativa*^27^. Gene sequences were used as queries for BLAST searches within closely related legume species such as *M. truncatula, C. arietinum* and *P. sativum*. Positive results were used to identify homologous sequences in an in-house faba bean transcriptome database developed by Ocaña et al.^69^. Primer design was run using Geneious software (v. 7.1.9; Biomatters, Auckland, New Zealand).

Orthologous gene sequences were aligned and when possible, primers were designed on *V. faba* sequences spanning an intron. Otherwise we used the sequence of related species to design primers with 20–25 nucleotides, GC content of 45–60%, and Tm of 60 ± 1 °C, yielding a PCR amplicon of 200–1000 bp. Markers developed in this study were named as the corresponding candidate gene preceded by the letters ‘Vf’ (Supplementary Table 3).

In addition to these markers, a set of 163 markers from the Kompetitive Allele Specific PCR (KASP) developed by Webb^33^, were selected and genotyped in this population (Supplementary Table 6).

### PCR amplification and polymorphism detection

Genomic DNA was isolated from young leaves using the CTAB procedure^70^. PCR amplification consisted of 25 μl reactions containing 4 ng template DNA, 1 × PCR buffer, 2 mM of MgCl_2_, 0.4 mM of dNTPs, 0.2 μM of each primer and 1 unit of Taq polymerase (Biotools B&M Labs, Madrid, Spain). Amplification conditions were as follows: initial denaturation at 94 °C for 5 min, followed by 30 cycles of 45 s at 94 °C, 45 s at 56–60 °C and 45 s at 72 °C, with a final extension step of 8 min at 72 °C. PCR products were separated using 2% agarose gels.

PCR amplification products lacking polymorphism between the parental lines were purified using a standard protocol (https://www.thermofisher.com) for DNA precipitation with sodium acetate and ethanol (1/10 3 M sodium acetate (pH 4.5), 2 v/v ethanol). Products were sequenced by Sanger at STABVIDA (Caparica, Portugal). Sequences were then aligned to detect SNPs using the Geneious software suite (v. 7.1.9; Biomatters, Auckland, New Zealand). Amplicons with recognition sites for restriction site polymorphism were converted into CAPS markers (Cleaved Amplified Polymorphism Sequence) to genotype the whole RIL population. Restriction digestions were performed following the supplier’s instructions and visualized in 2% agarose gels. For SNPs lacking restriction enzymes in the polymorphic site, internal primers were designed using the Tetra-Primer ARMS–PCR technique described by Medrano and Oliveira^71^. In those cases where the internal primers did not yield amplification, the genotyping was carried out at CEGEN-PRB3-ISCIII (https://www.cegen.org) using the MassArray iPLEX (Sequenom) SNP typing platform from the Spanish National Genotyping Center facility of the University of Santiago de Compostela.

### Genetic mapping and QTL analysis

Polymorphic markers genotyped in the RIL population were incorporated into a previous marker dataset^31^ to develop a new linkage map. Segregating data were analyzed for goodness of fit to the expected 1:1 ratio using the chi-squared tests. Linkage analysis was performed using JoinMap v4.0^72^ with the maximum likelihood option. Markers were grouped at a minimum LOD score of 3 and a maximum recombination fraction of 0.25. Recombination fractions were converted to centimorgans (cM) using the Kosambi mapping function^73^.

QTL analysis was conducted using MapQTL v5.0^74^. First, the nonparametric Kruskal–Wallis test was used to detect association between markers and traits. Then, interval mapping^75, 76^ was performed to identify putative QTLs in each linkage group (LG). Markers significant at *P* = 0.01 were used as cofactors in the multiple QTL analysis (MQM)^77–79^. QTL significance (*P* value) was determined by permutation analysis using 1000 replicates^80^, as implemented in MapQTL 5.0. Only QTLs with a LOD higher than the p-value were declared as significant. MapChart software^81^ was used to represent the QTLs confidence interval. The support intervals were defined as LOD-1 and LOD-2 around the maximum LOD of QTL.

### Histological sample preparation

**Figure 5:**
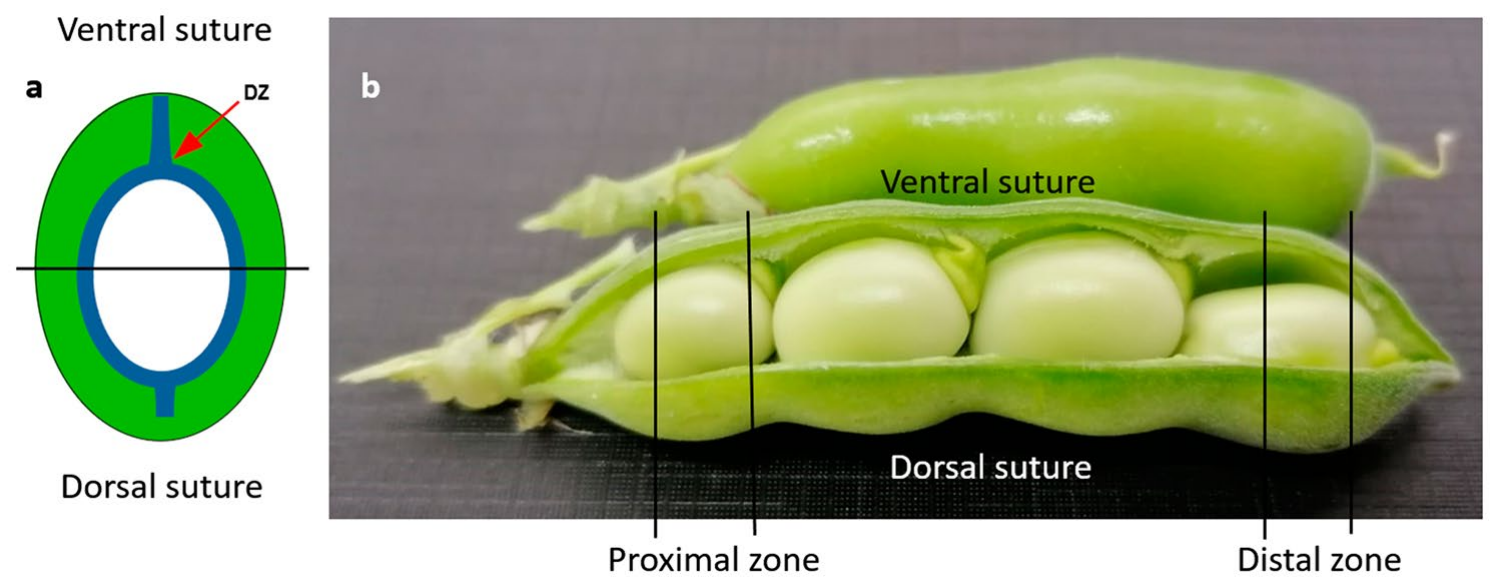
(**a**) Schematic representation of a pod transverse section. The blue line indicates lignin deposition. DZ, dehiscence zone. (**b**) Zones in pods where histological cuts were performed.

Plant material was prepared according to the protocol described by Pérez-de-Luque^82^. Mature pods (turning to brown) from parental lines were harvested and fixed in FAA (3.7% formaldehyde, 5% acetic acid and 50% alcohol). Proximal and distal edges were cut in transverse segments corresponding with the ventral and dorsal sutures of the pod (Fig. 5). Samples were dehydrated in an increasing series of ethanol/water solution from 50 to 100% of ethanol, 12 h each, then transferred to an embedding solvent through xylene-ethanol series (30, 50, 80, 100% twice, 12 h each) and finally samples were saturated with paraffin (Paraplast plus; Sigma, Switzerland). After saturation the solution was replaced with melted paraffin and the samples were kept at 56 °C for 24 h. This step was repeated twice in order to secure complete evaporation of xylene residues. Pod sections embedded in paraffin blocks were cut into 7 μm-thick sections using a Leica RM2245 rotary microtome (Leica Microsystems, Germany) and attached to adhesive treated microscope slides (polysine slides; Menzel GmbH and Co KG, Braunschweig, Germany).

### Staining procedure

Histochemical staining of the sections was carried out according to Ruzin^83^ with slight modifications^82^. Each slide was stained with 0.1% TBO (0.1 M citric acid and 0.1 M sodium citrate) for 20 min and washed with distilled water. Samples were then deparaffinized twice, with 100% xylene for 20 min and sealed with mounting medium (Entellan; Merck KGaA, Darmstadt, Germany). The sections were observed using a Nikon Eclipse 50i light microscope and photographs were taken through a Nikon DS-Fi1 digital optical device connected to a PC through the Nikon DS-U2 control unity (Nikon Instruments). Image analysis was performed using the ImageJ software^84^.

The area and perimeter of the lignified cell layer was measured in the left and right DZ regions. The percentage of lignin inside the cells was calculated as (total cell area—inner non-colored area)/total cell area × 100, and was measured in 20 cells.

## Supplementary information

Supplementary file1

Supplementary file2

## Data Availability

The datasets generated and analyzed during the current study are available from the corresponding author on request.
