# Supplementary material for: First approach to pod dehiscence in faba bean: genetic and histological analyses
Source: Sci Rep. 2020 Oct 19;10:17678. doi: 10.1038/s41598-020-74750-1 (PMC7572390; doi:10.1038/s41598-020-74750-1)

# First Approach to Pod Dehiscence in Faba Bean: Genetic and Histological Analyses

David Aguilar-Benitez[1] , Inés Casimiro-Soriguer[1] , Ana M. Torres[1]

[1] IFAPA center Alameda del Obispo, Apdo 3092, E-14080, Córdoba, Spain.

*Corresponding author e-mail: anam.torres.romero@juntadeandalucia.es

Supplementary Figure 1. Histological analysis of transverse sections from faba bean pods. Distal zone, dorsal suture in Vf6 (a) and Vf27 (b); proximal zone, ventral suture of Vf6 (c) and Vf27 (d); and distal zone, dorsal suture of Vf6 (e) and Vf27 (f). DZ, dehiscence zone; EP, epidermis; MS, mesocarp; VS, ventral sheath; DS, dorsal sheath; VB, vascular bundle; EN, endocarp.

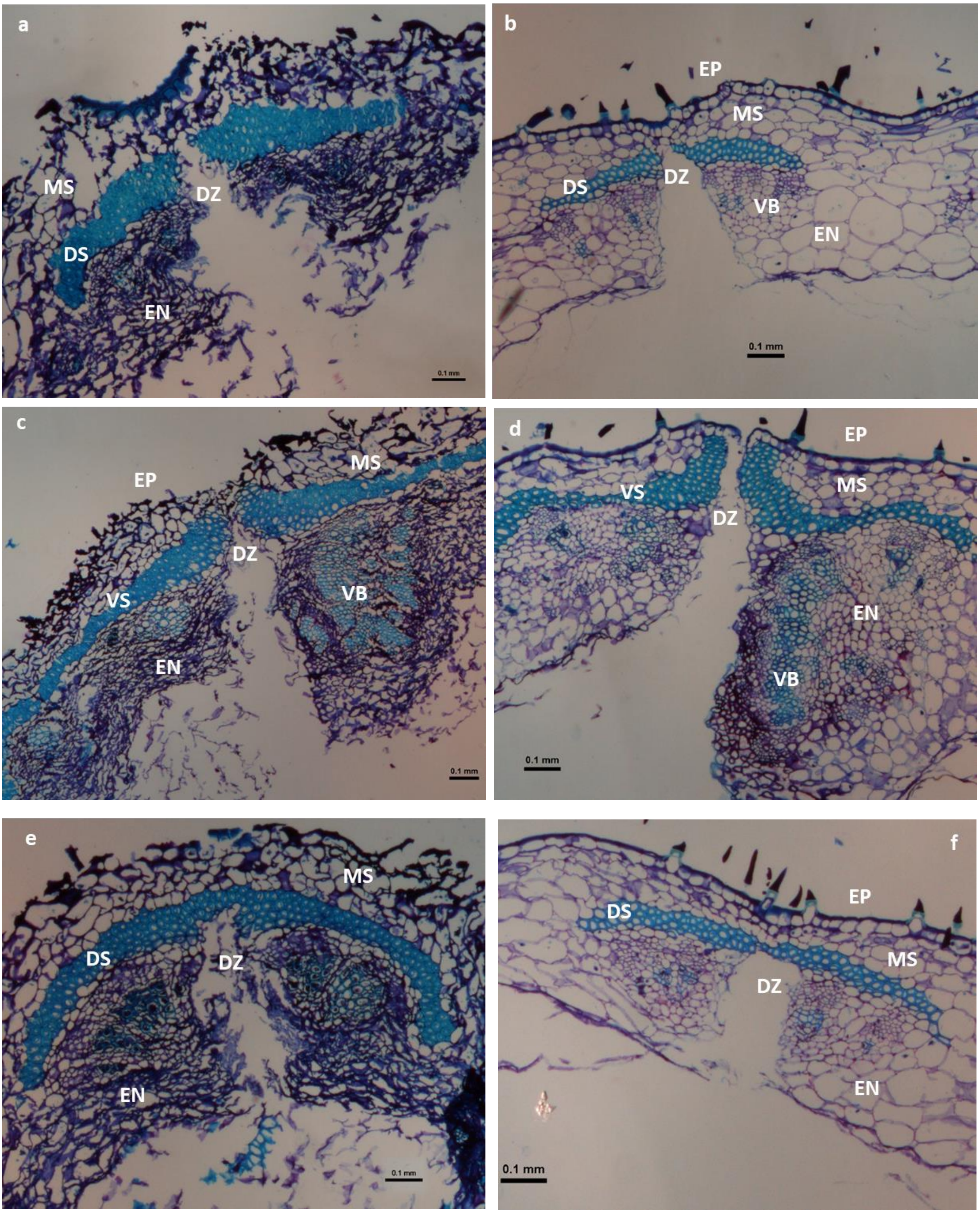

Supplementary Figure 2. Histological transversal cuts from dehiscent faba bean pods. Proximal zone, ventral suture in line 1068 (a); distal zone, ventral suture in line 335 (b); distal zone, dorsal suture in line 756-3 (c). Bars: 0.1 mm. DZ, dehiscence zone; EP, epidermis; MS, mesocarp; VS, ventral sheath; DS, dorsal sheath; EN, endocarp, LFL, lignified fibre layer.

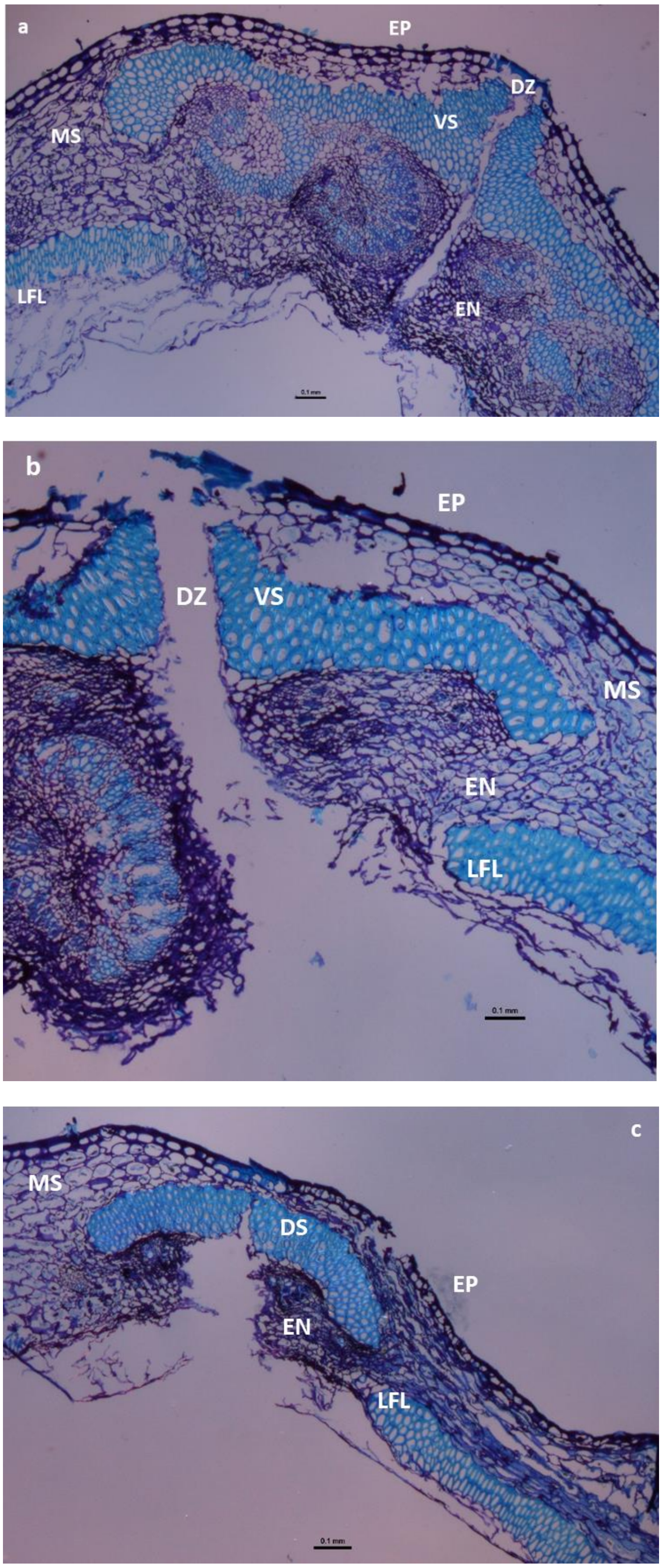

Supplement: Supplementary file 2 — Supplementary file2 [file 41598_2020_74750_MOESM2_ESM.pdf]
